# First report of *Planomicrobium okeanokoites* associated with *Himantothallus grandifolius* (Desmarestiales, Phaeophyta) from Southern Hemisphere

**DOI:** 10.1371/journal.pone.0282516

**Published:** 2023-04-14

**Authors:** Khem Chand Saini, Kriti Gupta, Sheetal Sharma, Ajay K. Gautam, Samrin Shamim, Divya Mittal, Pushpendu Kundu, Felix Bast

**Affiliations:** 1 Department of Botany, Central University of Punjab, Ghudda, Bathinda, Punjab, India; 2 Department of Botany, DAV College, Bathinda, Punjab, India; Central University of Kerala, INDIA

## Abstract

Gram-positive, aerobic, motile, rod-shaped, mesophilic epiphytic bacterium *Planomicrobium okeanokoites* was isolated from the surface of endemic species *Himantothallus grandifolius* in Larsemann Hills, Eastern Antarctica. The diversity of epiphytic bacterial communities living on marine algae remains primarily unexplored; virtually no reports from Antarctic seaweeds. The present study used morpho-molecular approaches for the macroalgae and epiphytic bacterium characterization. Phylogenetic analysis was performed using mitochondrial genome encoded COX1 gene; chloroplast genome encodes rbcL; nuclear genome encoded large subunit ribosomal RNA gene (LSU rRNA) for *Himantothallus grandifolius* and ribosomal encoded 16S rRNA for *Planomicrobium okeanokoites*. Morphological and molecular data revealed that the isolate is identified as *Himantothallus grandifolius*, which belongs to Family *Desmarestiaceae* of Order *Desmarestiales* in Class *Phaeophyceae* showing 99.8% similarity to the sequences of *Himantothallus grandifolius*, from King George Island, Antarctica (HE866853). The isolated bacterial strain was identified on the basis of chemotaxonomic, morpho-phylogenetic, and biochemical assays. A phylogenetic study based on 16S rRNA gene sequences revealed that the epiphytic bacterial strain SLA-357 was closest related to the *Planomicrobium okeanokoites* showing 98.7% sequence similarity. The study revealed the first report of this species from the Southern Hemisphere to date. Also, there has been no report regarding the association between the *Planomicrobium okeanokoites* and *Himantothallus grandifolius*; however, there are some reports on this bacterium isolated from sediments, soils, and lakes from Northern Hemisphere. This study may open a gateway for further research to know about the mode of interactions and how they affect the physiology and metabolism of each other.

## Introduction

Antarctica is one of the coldest, darkest, driest, and windiest continents on the Earth. The large ozone hole, high range of UV radiations, and dark winters for six months attribute to less biodiversity in this region [1]. During summers, only 0.3% area is free from ice. Oases, coastal outcrops, offshore islands, mountain ranges, and nunataks are several ice-free areas where life in terms of flora and fauna is confined [2]. To understand the survival of living beings in this cold environment, several expedition programs were conducted. The first expedition was carried out in 1981–82 by Indian researchers to Schirmacher Oasis and Queen Maud Land, Antarctica. Later on, Indian explorers started surveying areas around an Indian station named Bharati at Larsemann Hills, Eastern Antarctica. The diversity in Antarctica is rich in terms of marine alga and high endemism despite the harsh environmental conditions. The benthic flora of Antarctica comprises 117 species (16 Chlorophyta, 27 Ochrophyta, and 74 Rhodophyta), with 57 species endemic to Antarctica [3]. Recent studies revealed that Antarctica is turning ’green’; because of increased sporadic algal blooms and mosses in response to global climate change [4, 5]. So, the assessment of floristic diversity in this region becomes critical.

*Himantothallus* is a genus of brown alga that currently includes a single species viz *grandifolius*. This species is confined to the Southern Hemisphere and endemic to Antarctica and South Georgia [6]. It is one of Antarctica’s most common and widely distributed seaweeds [7, 8]. It is a giant, kelp-like plant consisting of a single to several undivided strap-shaped thick blades which can measure up to 10 m in length and 1 m in breadth, born on a very short twisted flat stipe and anchored by a hapteroid holdfast. Its blades can grow up to 14m and a half-meter wide [9]. The degree of endemism is higher in the Antarctic marine flora [10], with utmost levels in the *Heterokontophyta* and the *Rhodophyta*. This macroalgae is adapted to the low seawater temperatures and usually grows at 5°C, forming a suitable habitat for epiphytic bacteria growing on macroalgal species. The alga can grow singly, in small clusters, or dense strands in the Antarctic waters and on the Scotia Arc Islands (South Atlantic Ocean), where they are widespread and represent the dominant portion of benthic seaweeds [7, 11]. They are attached to rocky substrates, mostly in subtidal habitats forming extensive belts. The taxonomic entity of *Himantothallus grandifolius* needs to be confirmed from Larsemann Hills, Eastern Antarctica, for which this study attempts phylogenetic analysis.

Macroalga from Antarctica is often associated with microbial populations that differ significantly in tropical and temperate waters [12]. The surface of marine macroalgae forms an endurable habitat for microbial colonization. Few studies have been reported about the Gram-positive bacterial communities in terms of diversity when they are associated with Antarctic marine macroalgae. Bacteria are the primary dominant colonizers of algal surfaces in terms of space,occupation, and abundance [13]. Several studies reported differences between the macroalgae’s surface inhabited by microbial populations in surrounding seawater [14–17]. Each algal host provides a distinct ecological niche with unique abiotic and biotic characteristics.

Some culture-dependent research works have visualized the pigmented widely distributed bacterial community in the marine environment [18]. Bacteria with orange and yellow pigmentation are frequently inhabitants on the surfaces of marine macroalgae. These pigmented bacteria absorb the UVA-blue and visible spectral lights in these marine environments [18]. However, the diversity, distribution, and ecological functions of Antarctic Gram-positive heterotrophic bacterium remain unclear. Few studies have explored the diversity, distribution, and ecological significance of the epiphytic bacteria related to Antarctic macroalgae compared to their terrestrial relatives [19].

Nakagawa et al. [20] transferred *Flavobacterium okeanokoites* to genus *Planococcus* as *Planococcus okeanokoites*. Later on, Junge et al. [21] isolated three-Gram positive bacterial strains from brine samples of the sea ice community in Antarctica, which they described as a new species *Planococcus mcmeekinii*. After this study, Yoon et al. [22] first proposed the genus *Planomicrobium* and transferred *Planococcus okeanokoites* to *Planomicrobium okeanokoites* in this genus. In 2017, Barreto et al. [23] isolated four bacterial strains viz. *Marinomonas* sp., *Pseudomonas* sp., *Pseudoalteromonas* sp., and *Sulfitobacter* sp. from the surface of *H*. *grandifolius* collected at King George Island, Antarctica. The species earlier reported being from various habitats such as fermented seafood, marine mud, salt lakes, cold desert soils, intertidal sediments, and glaciers. So far, *Planomicrobium* has comprised eleven species: *Planomicrobium alkanoclasticum* [24, 25]; *Planomicrobium chinense* [25]; *Planomicrobium flavidum* [26]; *Planomicrobium iranicum* [27]; *Planomicrobium koreense* [22]; *Planomicrobium mcmeekinii* [21, 22]; *Planomicrobium okeanokoites* [22, 28]; *Planomicrobium psychrophilum* [25, 29]; *Planomicrobium soli* [30]; *Planomicrobium stackebrandtii* [26, 31] and *Planomicrobium alkanoclasticum* [25]

The present study attempts the morpho-phylogenetic, chemo-taxonomic characterization of the Gram-positive bacterium i.e., *Planomicrobium okeanokoites* isolated from macroalgae *Himathothallus grandifolius* of Larsemann Hills, Eastern Antarctica. For phylogenetic analysis of macroalgae and bacteria, we employed Mitochondrial cytochrome c oxidase (cox1), chloroplast Ribulose-1,5 bisphosphate, and nuclear- Large ribosomal subunit (LSU rRNA) for macroalgae and 16S rRNA sequences for bacteria. According to the literature survey, *P*. *okeanokoites* were not earlier reported from Southern Hemisphere as per our knowledge. However, some studies attempt its taxonomy and species transfer from one genus to another.

## Material and methods

### Sample collection

The isolates of *Himantothallus grandifolious* collected from Larsemann Hills, Eastern Antarctica (69˚22ʹ24.6ʺS 76˚13ʹ41.9ʺE) during the 36^th^ Indian Scientific Expedition to Antarctica (ISEA) in 2016–2017. Samples were packed in sterile plastic zip-lock bags and stored at -80°C for further studies. The representative specimen was pressed and deposited in the herbarium of Central University of Punjab, Ghudda, Bathinda, India, with voucher number WP-906: CUPVOUCHER-HiGr-2019-1.

### Morphological examination of the algae

The samples were carefully washed in filtered sterile seawater (FSSW). Photographs were taken by using a bright-field microscope (BX53, Olympus, Japan), a digital SLR camera with Canon macro lens (EOS 60D, Japan). Analysis of the samples was done on the basis of their colour, texture, length of the thallus, shape, and arrangement of the cells. Public domain software ImageJ (http://rsbweb.nih.gov/ij/) was used for scale calibration and size measurements.

### Isolation, cultivation, and biochemical characterization of the bacterium

The fronds were rinsed three times with filtered sterile seawater (FSSW) to remove loosely attached microbes and cut them into small pieces. The epiphytic bacterial strain was isolated through serial dilution in FSSW and plating techniques. The macroalgae surface was rubbed with a sterile cotton swab, and the extracted bacteria present in the cotton swab were inoculated in marine agar and marine broth 2216 (DIFCO). The inoculated plates were incubated at 10°C and regularly inspected for growth for up to 4 weeks. Distinct colony morphotypes were restreaked on fresh medium until pure cultures were obtained based on their morphological characteristics by successive streaking using the ZoBell Marine Agar media (Himedia). The isolated bacteria were then observed for size, pigmentation, form, shape of the margins, and colony. The bacterium culture was used to observe the motility and Gram staining, which was confirmed through a low-power (10X) and high-power objective (40X) microscope. The bacterial strain was also examined for catalase, oxidase, and starch hydrolysis tests using standard protocols.

### Molecular identification of isolates and phylogenetic analysis

#### DNA extraction, amplification, sequencing

Genomic DNA from epiphytic bacterial strain was extracted using HiPurA^TM^ Bacterial genomic DNA extraction kit, and algal DNA was extracted using HiPurA^TM^ Marine Algal DNA Purification Kit (HiMedia Laboratories Pvt. Ltd., Mumbai). The concentration of DNA was checked on a Nanodrop spectrophotometer. The 16S rRNA gene sequences were amplified by using the universal primers 27F (5'AGAGTTTGATCMTGGCTCAG-3') and 1492R (5’TACGGTTAACCTTGTTACGACTT-3') [32], The algal DNA were amplified by using universal *rbcL* (RuBisCO Large-subunit) [33, 34], *cox1* (cytochrome oxidase subunit1) [35], and LSU rRNA [36] primers sets with DreamTaq™ DNA Polymerase (Applied Biosystems, Foster City, CA, USA). PCR amplifications were performed in programmable thermal cyclers (Bio-Rad Laboratories) with initial denaturation at 95°C for 3 minutes followed by 30 cycles of denaturation, annealing at 95°C for 1 minute and 55°C for 1 minute with final elongation step at 72°C for 7 minutes [37]. The final PCR products were analyzed by 1.2% Agarose gel electrophoresis. The amplified sequences were purified using an ExoSAP-IT® PCR clean-up kit (USB Corporation, Cleveland, OH, USA) to avoid downstream interventions.

Purified PCR amplicons were subjected to bi-directional sequencing PCR using ABI BigDye Terminator Cycle Sequencing Ready® Reaction Kit v3.1 (Applied Biosystems, Foster City, CA, USA). Sequencing reactions were purified using the traditional ethanol/EDTA precipitation method [38]. The dried samples were suspended again in 15 μl of Hi-Di™ Formamide and vortexed for 30 minutes, and then transferred to a sequencing plate for capillary gel electrophoresis (Applied Biosystems 3730 xl Genetic Analyzer, Foster City, CA, USA).

#### Sequence annotation and phylogenetic analysis

The sequence analysis and contig assembly were performed using licensed bioinformatics software Geneious® prime v2020.0.4 (Biomatters Limited, New Zealand, available at https://www.geneious.com). The rbcL, COX1, and LSU sequences of algae and 16S rRNA sequences of bacteria were base call and annotated carefully. For sequence homology search BLASTn (www.blast.ncbi.nlm.nih.gov) was used. The newly generated sequences of *Himantothallus grandifolius* (accession no. MT274692, MZ676777, and MZ613320) and *Planomicrobium okeanokoites* (accession no. MT275689) were deposited in the NCBI Genbank database. Multiple sequence alignments were carried out using 16S rRNA gene sequences of the isolate, and other reference sequences were downloaded from the NCBI database (Table 3). These were aligned by the MUSCLE algorithm [39]. The end of aligned sequences was trimmed to reduce the number of missing sites across taxa and was aligned by MUSCLE algorithm in Geneious Prime.

Phylogenetic analysis of bacteria was conducted using Maximum Likelihood (ML) and Bayesian Inference methods. In Bayesian phylogenetic inference, MrBayes v3.2.6 plugin [40] was selected, followed by pairwise distance calculation. Pairwise distances between sequences of the samples were calculated using the nucleotide substitution test model in Geneious^®^ Prime v2020.0.4. Bayesian analyses using the Markov chain Monte Carlo (MCMC) [41] technique was performed by MrBayes v3.2.6. The MCMC chains included four heated chains with 0.5 heated chain temperature and 200 subsampling frequencies. The Hasegawa-Kishino-Yano model [42] was used with a gamma-distributed variation 16S rRNA gene dataset and initiated an analysis from a random starting tree run for one million generations. The Maximum Likelihood method was performed using PhyML 3.3 in Geneious^®^ Prime, and substitution bias was modelled by the Hasegawa-Kishino-Yano model with a gamma-distribution. A total of 1000 bootstrap replicates were examined under the ML criterion to estimate interior branch support. In the phylogenetic analysis, 17 nucleotide sequences were involved, and *Bacillus subtilis* ATCC 21331 (AB018487) was used as an out-group [43].

### Results and discussion

According to the current understanding, the Antarctic macroalgae ecology appears to be hindered by the limited available database. In particular, a significant part of the Eastern Antarctic Coast between 45°E and 160°E is certainly under-sampled. Epiphytic bacteria that grow on the macroalgae surfaces live in a healthy competitive environment with limited space and access to nutrients. Many records are based solely on dredged or drift specimens, which are of limited usefulness or are doubtful because they were sampled only very few times and could have been confused with morphologically similar species. However, this is low species diversity relative to the world’s temperate and tropical regions, but in a similar range as in the Arctic [44]. Heterokontophyta and Rhodophyta were found plenty in the marine environment. This research concerned isolating and detecting macroalgae-related epiphytic bacteria from Larsemann Hills, Eastern Antarctica. In this study, a strain of marine bacterium *Planomicrobium okeanokoites* associated with brown algae *Himantothallus grandifolius* was isolated. The present study made several fascinating revelations about the epiphytic and phylogenetic relationships between gram-positive bacteria and Antarctic macroalgae, as little information is available to date.

### Morphological analysis of macroalgae

**Fig 1:**
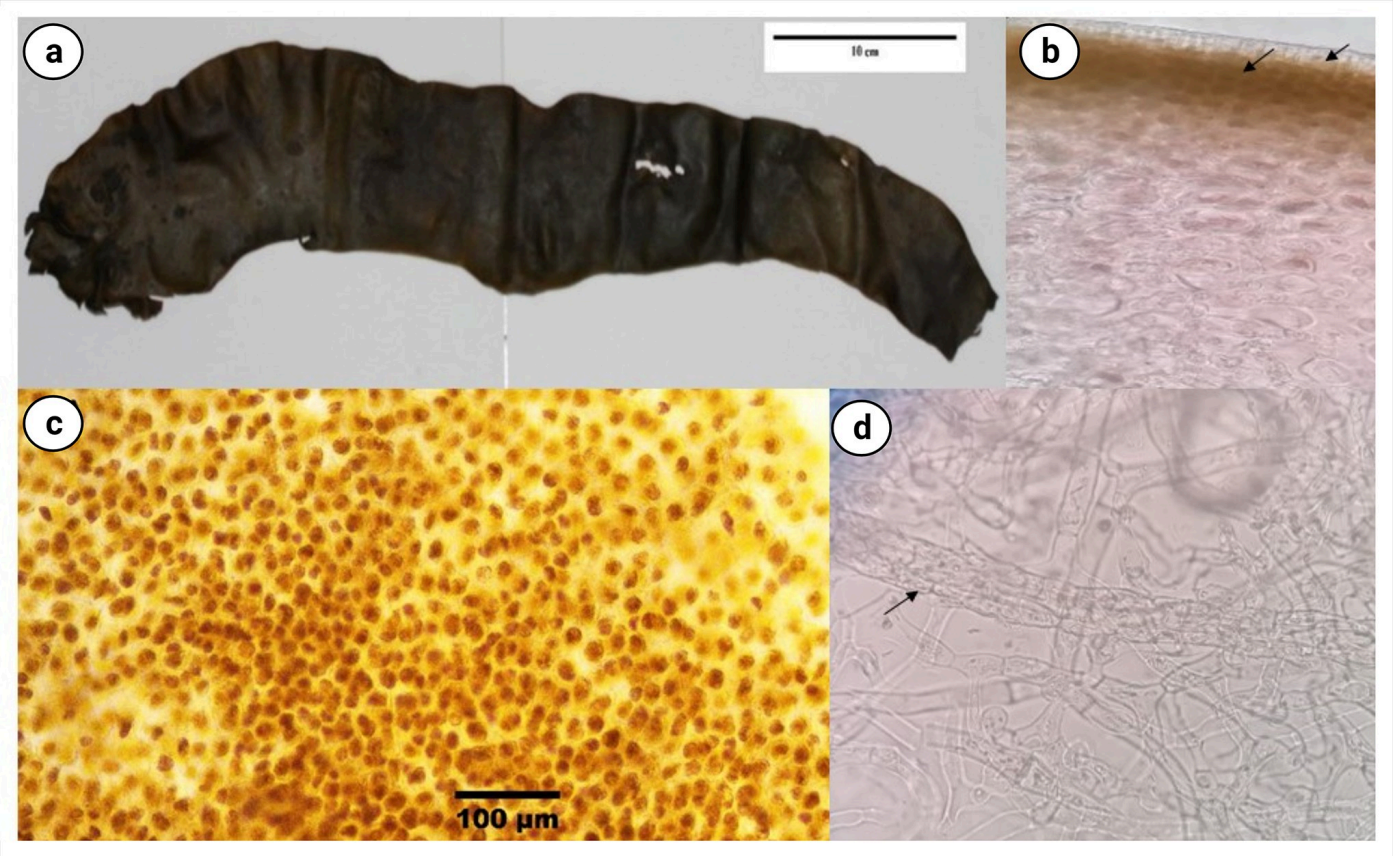
Morphological images of *Himantothallus grandifolius*. (a) External morphology of the thallus. (b) Margin of the blade showing meristoderm (inner arrow), subtending cortical cells (outer arrow), and a network of cortical filaments (40X). (c) Cells arrangement (10X). (1d) Plexus of unsheathed filaments intermingled with sheathed trumpet hyphae (arrow) that run in a longitudinal direction (40X).

The thallus of the *Himantothallus grandifolius* appears dark-light brown, thick, and leathery, measuring around 45–50 cm in height, and is strap-shaped with ruffled margins (Fig 1A). The blades were tapered towards one end. Anatomically, the blade’s anatomy shows separation across three layers. The outermost layer, meristoderm, is one cell thick, composed of rectangular cells deficient in physodes (Fig 1B). It is strap-shaped with ruffled margins (Fig 1A). The middle layer cortex consists of densely packed, sub-spherically shaped, parenchyma-like cells which contain physodes. The cells are very densely packed (Fig 1C). The inner layer, the medulla, consists of plexus of rectangular cell filaments, which sometimes dichotomize (Fig 1D). The appearance of sheathed trumpet hyphae is a characteristic feature of *Himantothallus*, which is also seen in running longitudinally (Fig 1D).

### Molecular analysis of macroalga

This study generated sequence information of *Himantothallus grandifolius* at Mitochondrial cytochrome c oxidase (cox1), chloroplast Ribulose-1,5 bisphosphate, and nuclear- Large ribosomal subunit (LSU rRNA) region. The sequences were submitted to GenBank, and the Accession numbers for rbcL, COX1, and LSU are MT274692, MZ676777, and MZ613320, respectively. NCBI top BLASTn hits for three loci are presented in Table 1. From this, we inferred that the specimen is *Himantothallus grandifolius* which belongs to the family Desmarestiaceae of Order Desmarestiales in Class Phaeophyceae.

**Table 1:** Top BLASTn hits of the macroalgal sequence.

S. No.	Primers Sequences of isolate	Closest species as per NCBI BLASTn hits (Accession Numbers)	Location	% Identity
1	rbcL	*Himantothallus grandifolius* (HE866853)	King George Island, Antarctica	99.85%
2	COX1 (615)	*Himantothallus grandifolius* (MK503231)	Isla Sapo, O’Higgins, Antarctica	97.08%
3	Large subunit ribosomal RNA gene (LSU)	*Himantothallus grandifolius* (MF419237)	King George Island, Antarctica	99.67%

### Morphological and biochemical characterization of an isolated bacterium

**Fig 2:**
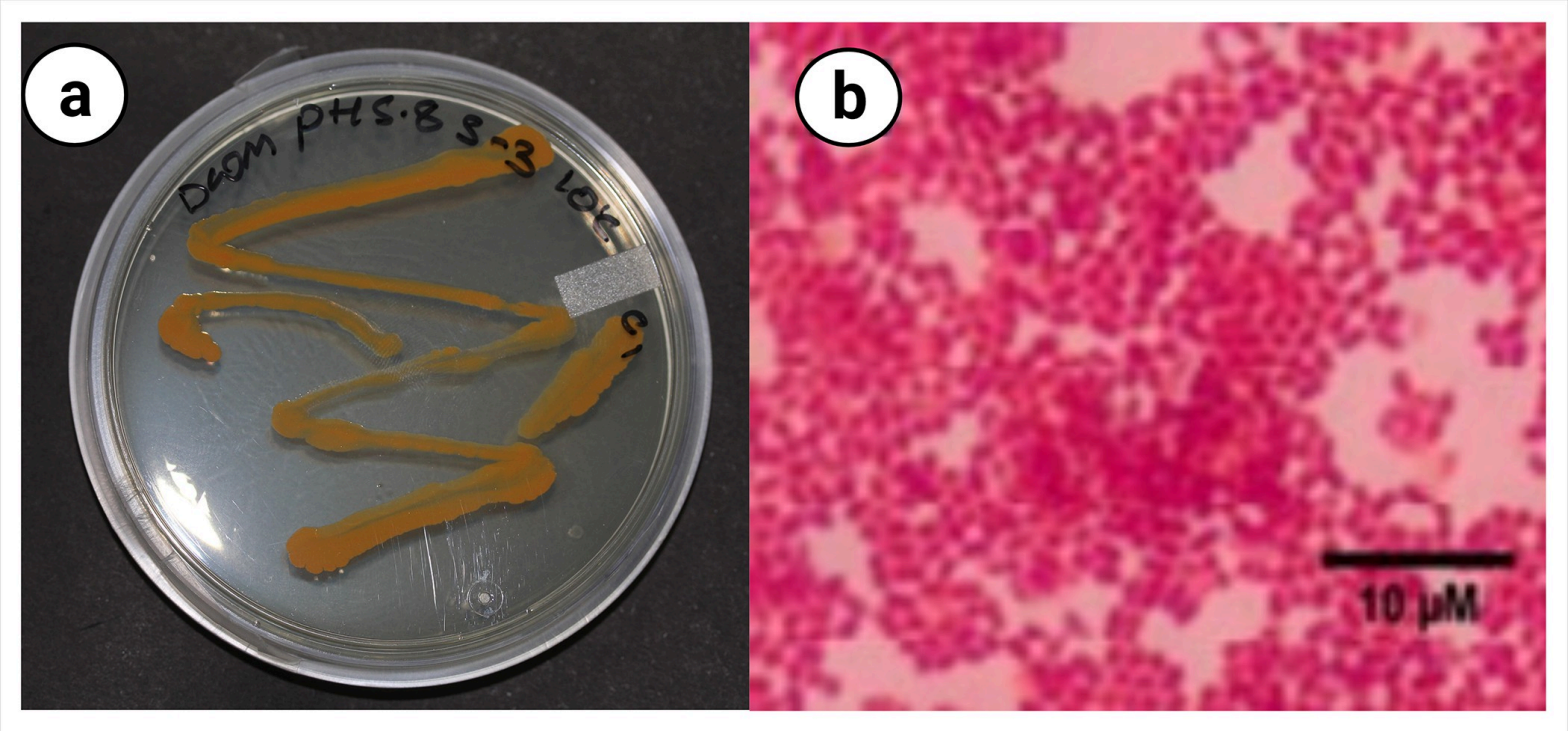
**(a)** The morphological features of strain analysed in this study **(b)** Stained bacterial cells of the isolates.

The isolated epiphytic bacterial strain with colonies bearing pale yellow to orange in colour (Fig 2) and rod-shaped motile cells. The biochemical assay showed a positive response for catalase, urease, and oxidase and negative for the starch hydrolysis test. The comparative results of biochemical determinations with different species of *Planomicrobium* with new epiphytic bacterial strain showed in Table 2.

**Table 2:** Differential phenotypic characteristics of various *Planomicrobium* species.

Characteristics	*P*. *flavidum*	*P*. *koreense*	*P*. okeanokites*	*P*. *mcmeekini*	*P*. *chinense*	*P*. *psychrophilum*	*P*. *alkanoclasticum*	*P*. *glaciei*	*P*. *sttackebrandtii*
**Gram Reaction**	+	+	**+**	+	+	+	+	+	+
**Cell morphology**	C or SR	C or SR/R	**R**	R/C or SR	C or SR	R	R	C or SR	C
**Motility**	+	+	**+**	+	+	+	+	+	+
**Pigmentation**	Light yellow	Yellow orange	**Yellow orange**	Pale orange	Yellow orange	Orange	Orange	Yellow orange	Orange
**Oxidase**	+	-	**+**	-	-	+	-	-	-
**Catalase**	+	+	**+**	+	+	+	+	+	+
**Hydrolysis of starch**	-	-	**-**	-	-	-	+	-	-

C = Coccoid; SR = Short rod; R = Rod shaped; (+) positive = (-) negative

*The strain of epiphytic bacterium isolated from *Himantothallus grandifolius*.

### Phylogenetic analysis of epiphytic bacterium

Phylogeny of the 16S rRNA sequence with additional 16 nucleotide sequences were involved in constructing a phylogenetic tree where *Bacillus subtilis* ATCC 21331 (AB018487) was used as an out-group. For phylogenetic analysis, sequences were first aligned by the MUSCLE algorithm in MEGA. In clade one, the isolate clustered with other *P*. *okeanokoites* strains of related taxa procured from the NCBI database with 1.00 PP/97% bootstrap values. Different reports on *Planomicrobium* sp. with their isolation sources/hosts are given in Table 3.

**Table 3:** Accession numbers of 16S rRNA sequences procured from the NCBI database.

Sr. No.	Taxon	Isolation source/host	Location	16S rRNA
**1**	***Planomicrobium okeanokoites***	***Himantothallus grandifolius***	**Eastern, Antarctica**	**MT275689 (generated in this study)**
**2**	*Bacillus subtilis* ATCC 21331	-	-	AB018487
**3**	*P*. *chinense* DX3-12	Coastal sediment	China	AJ697862
**4**	*P*. *glaciei* 0423	Frozen soil	China	EU036220
**5**	*P*. *flavidum* ISL-41	Marine solar saltern	South Korea	FJ265708
**6**	*P*. *stackebrandtii* JCM 12481	Cold desert soil	India	LC076757
**7**	*P*. *okeanokoites* YT184	Sediment	China	MH725427
**8**	*P*. *okeanokoites* DAS41	Soil	South Korea	MH819716
**9**	*P*. *okeanokoites* DAS47	Soil	South Korea	MH819721
**10**	*P*. *okeanokoites* wp-11	Salinized soil	China	MK610679
**11**	*B*. *cereus* AR2019-1	Soil	India	MN148885
**12**	*P*. *okeanokoites* SLA-335	Salt Lake	China	MT125773
**13**	*P*. *okeanokoites* SLA-357	Salt Lake	China	MT125787
**14**	*P*. *stackebrandtii* K22-03	Cold desert	India	NR025781
**15**	*P*. *chinense* DX3-12	Coastal sediment	China	NR042259
**16**	*P*. *glaciei* 0423	Frozen soil	China	NR044384
**17**	*P*. *flavidum* ISL-41	Marine solar saltern	South Korea	NR116601

**Fig 3:**
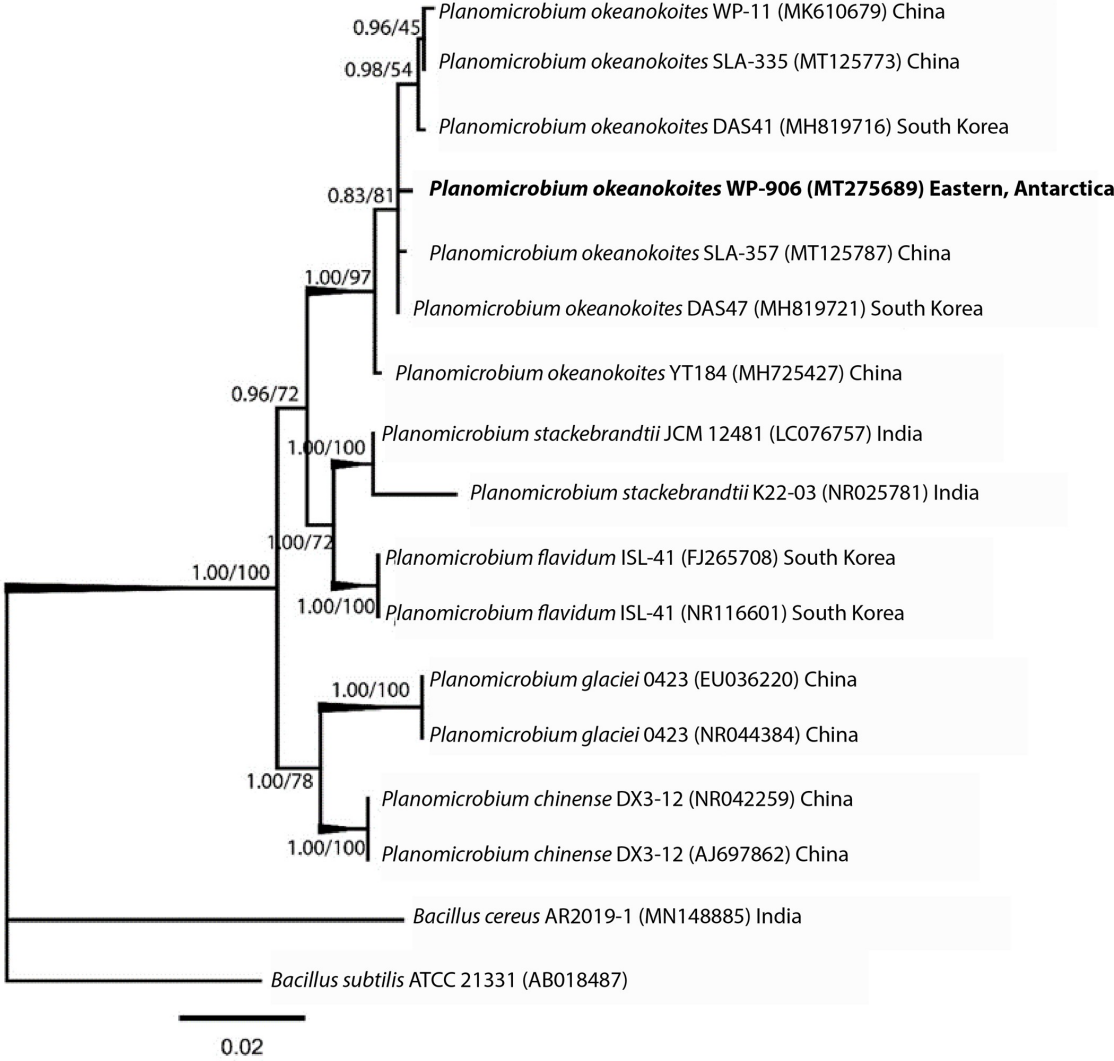
Phylogenetic tree based on the 16S rRNA gene of bacterial isolates constructed by Bayesian inference and maximum likelihood methods (The sequenced *P*. *okeanokoites* gene is marked in bold).

The NCBI BLASTn searches of 16S rRNA gene sequences of isolated epiphytic bacterial strain showed the best matches (based on percentage identity) >98.78% with *Planomicrobium okeanokoites* SLA-357 (MT125787). Top ten BLASTn hits of the bacterial sequence showed approx. 98% similarity to the isolated epiphytic bacterial sequence (Table 4). 16S rRNA gene sequence of epiphytic bacterium generated in this study was 1394 base pair (bp) in length. A phylogenetic tree based on 16S rRNA gene sequences was constructed using the maximum-likelihood (ML) and Bayesian inference methods (Fig 3). Values at the nodes indicate posterior probability support and bootstrap values. Full statistical posterior probability support (1.00 PP/100% PP). The highly supported branches are shown in bold (Posterior Probability > 0.95 calculated with MrBayes and bootstrap values >95 using maximum likelihood).

**Table 4:** Top 10 BLASTn hits of the bacterial sequence.

S. No.	Species	Location	GenBank Accession number	% Identity
**1**	*Planomicrobium okeanokoites* SLA-357	China	MT125787	98.78%
**2**	*Planomicrobium* sp. Y7	China	EF177691	98.78%
**3**	*P*. *okeanokoites* DAS41	South Korea	MH819716	98.71%
**4**	*Planomicrobium* sp. R20	-	KM017984	98.64%
**5**	*P*. *okeanokoites*	China	MH725427.	98.64%
**6**	Bacterium MTL5-52	China	MH151236	98.64%
**7**	*P*. *okeanokoites* DAS47	South Korea	MH819721	98.78%
**8**	*P*. *okeanokoites* NF22B	China	MT269281	98.64%
**9**	*P*. *okeanokoites* NF22A	China	MT269279	98.64%
**10**	*P*. *okeanokoites* SLA-335	China	MT125773	98.64%

From morpho-phylogenetic studies, it can be concluded that the isolated bacterium is *Planomicrobium okeanokoites* which provides new insights into understanding the epiphytic associations between bacterial strains and the algal microbiome in Antarctica. Macroalgae provide nutrients and shelter to bacteria as a by-product of their photosynthesis, whereas bacteria provide vitamins, such as Vit B_12_ [45] and growth factors [46] for algal growth [47, 48]. Such intimate epiphytic associations proved macroalgae and bacteria as a holobiont or unified functional entity. The analysed data have been validated with the isolated bacterium *Halimeda* sp. associated with green algae from Lake Kakaban, Indonesia [49]. This research was also validated by three Antarctic subtidal macroalgae (*Himantothallus grandifolius*, *Pantoneura plocamioides*, and *Plocamium cartilagineum*) by a similar study, two of them were investigated as a source for isolation of agar-degrading bacteria, identified based on 16S rRNA belonged to the genera *Cellulophaga*, *Colwellia*, *Lacinutrix*, *Olleya*, *Paraglaciecola*, *Pseudoalteromonas* and *Winogradskyella* [50].

### Conclusion and prospectives

This study gives a first report on the presence of a gram-positive bacterium *Planomicrobium okeanokoites*, from the surface of *Himantothallus grandifolius* (Desmarestiales, Phaeophyta), from Larsemann Hills, Eastern Antarctica. In addition, this is the first report of *Planomicrobium okeanokoites* from the Southern Hemisphere to the best of our knowledge. This study is based on the chemotaxonomic and morpho-phylogenetic identification of *Planomicrobium okeanokoites* from *Himantothallus grandifolius*. However, there are some reports on this bacterium from sediments, soils, and lakes from the Northern Hemisphere. This study has a taxonomic importance and provides insights into understanding the origin of life of the bacterium in a very harsh climate conditions. This species might be introduced by some means or may be already existing there. Some studies have described the role of an epiphytic bacterium in influencing the metabolism and morphology of the host plant [51, 52]. For example, a bacterium associated with *Ulva mutabilis* is responsible for the development of blade morphology, its adhesiveness to the substratum, and growth [53]. This study may open a gateway for further research to know about the mode of interactions and how they affect the physiology and metabolism of each other. This study sets a prospect to understand how bacterial strains are associated with the algal microbiome in Antarctica and the critical processes involved in the association. However, this study revealed the identification of the bacterium *Planomicrobium okeanokoites* from Larsemann Hills, Eastern, Antarctica on the *Himantothallus grandifolius* (Desmarestiales, Phaeophyta). However, future research is needed to infer the compounds involved in epiphytic association of the bacterium *Planomicrobium okeanokoites* with *Himantothallus grandifolius*.
